# What is Sympathy? Understanding the Structure of Other-Oriented Emotions

**DOI:** 10.1177/17540739221140404

**Published:** 2022-11-21

**Authors:** Elodie Malbois

**Affiliations:** Institute for Ethics, History, and the Humanities, 28535 University of Geneva, Geneva, Switzerland

**Keywords:** sympathy, empathy, empathic concern, emotions

## Abstract

Sympathy (empathic concern) is mainly understood as a feeling *for* another and is often contrasted with empathy—a feeling *with* another. However, it is not clear what feeling *for* another means and what emotions sympathy involves. Since empirical data suggests that sympathy plays an important role in our social lives and is more closely connected to helping behavior than empathy, we need a more detailed account. In this paper, I argue that sympathy is not a particular emotion but a type of emotional experience: those that have another person as focus. I explain what this means and show that this sheds light on why sympathy, rather than empathy, directly motivates altruistic actions.

Empathy and sympathy, which is also called empathic concern,^1^ involve affective states that are more appropriate to another person's situation than our own. As I will understand them here, the difference between them can be broadly understood as one between feeling *with* and *for* another (Slote, 2007, p. 13). Empathizing consists in feeling what another is feeling, or something close enough, often as a result of taking that person's perspective, although not necessarily (de Vignemont & Singer, 2006; Maibom, 2017). Sympathy is a reaction from a caring third-person perspective (Darwall, 1998). It involves feeling happy for another when something good happens to her and sad for her when something bad happens to her (Maibom, 2017, p. 2). If a friend is going wingsuit flying, for instance, empathizing with her might include putting ourselves in her shoes and feeling her thrill about doing it, while sympathizing with her might make us feel worried about her because wingsuit flying looks very dangerous to us.

Although this distinction between empathy and sympathy is often put forward, most authors only provide a broad characterization of sympathy. As a result, it is not clear what emotions can be felt in sympathy (Maibom, 2014). According to Batson, when felt for someone in a difficult situation, sympathy can include sadness, worry, sorrow, compassion, and warmth among others (2011). Furthermore, while authors agree that sympathy involves concern for another, they do not specify if concern is part of the emotional experience of sympathy, occurring along with it or causing it. Our current understanding of this affect seems to rely mostly on the intuitive understanding of the distinction between feeling *with* and *for* someone.

The lack of precise understanding of what characterizes sympathy has not prevented empirical research on it. A growing body of research in psychology suggests that sympathy, rather than empathy, directly motivates altruistic behavior (Batson, 2011). Given the important role that sympathy is taken to play in our social lives, we need a more precise understanding of this emotional phenomenon. The intent of this paper is to remedy this lack. Using the notion of *focus* of an emotion from theories of emotions (Helm, 2007), I argue that sympathy is not an emotion but a type of emotional experience and that it refers to all instances of emotion that have another person as focus. This account clarifies the structure of sympathy, which emotions it involves and how the concern is woven into the experience. It also sheds light on why sympathy is more closely related to altruistic behavior than empathy.

In the first part, I review existing accounts of sympathy. I show that although there is a common broad understanding of sympathy, it lacks precision and there is disagreement regarding what emotions it includes. In the second part, I argue that emotions *for* others are emotions that have another person as their focuses. I explain the notion of focus of an emotion and show how it helps us understand why other-oriented emotions involve concern for another and motivate helping behavior. In the third part, I explain why we should understand sympathy as referring to all instances of emotion that have another person as focus. I end by contrasting the affective structure of sympathy with the one of empathy which illuminates the difference between them and makes it readily understandable why sympathy motivates helping actions more directly than empathy.

## The Current Understanding of Sympathy

While Hume and Smith already used the term “sympathy,” they understood it in ways that are closer to what we would call today “empathy” (see Section 4) (Hume, 1738; Smith, 2010). Scheler made an important contribution to what we will call “sympathy” here by distinguishing it from other emotional experiences such as emotional contagion (2017).^2^ According to him, sympathy consists in commiserating with another in pain and feeling pity/sorry for her (*Mitleid*) or in rejoicing with her happiness (*Mitfreude*) (Scheler, 2017). ^3^ Importantly, Scheler specified that when we sympathize with another, we do not feel that person's emotion and we do not take her perspective.

Current accounts of sympathy are in line with Scheler's: sympathy is understood as a feeling *for* another. It is different from empathy which is a feeling *with* another that involves emotional sharing and perspective taking. More precisely, authors like Darwall, Eisenberg, Coplan, and Maibom appear to agree with Batson that sympathy consists in feeling an other-oriented emotional reaction “elicited by and congruent with the perceived welfare of someone else” (Batson, 2011, p. 11). Other-oriented emotions have a positive valence (e.g., happiness, joy, excitement) when the other's welfare is perceived as positive, and a negative one (e.g., sadness, worry, anger) when the other's welfare is perceived as negative. This includes feeling sorry for someone who is in pain and happy for someone who has just reached a long-term goal. By contrast, it does not include schadenfreude since it is a positive emotional reaction to someone's difficulty. There is an array of emotions that can satisfy the above characterization of other-oriented emotions including feeling embarrassed, angry, concerned, sad, happy, and joyful for another. Batson, Darwall, Eisenberg, Coplan, and Maibom do not agree whether sympathy refers to all other-oriented emotions, some of them or only one of them.

Batson restricts sympathy, or “empathic concern”^4^ as he calls it, to other-oriented emotions felt for someone in need. It implies that for him, sympathy involves only emotions with a negative valence like “compassion, softheartedness, tenderness, sorrow, sadness, upset, distress, concern, and grief” (Batson, 2011, p. 11). Einsenberg, whose definition of sympathy is also used by Coplan, further restricts sympathy to “feelings of sorrow or concern for the other” (Eisenberg & Eggum, 2009, p. 72; Coplan, 2004). This restriction of sympathy to emotions felt for another in need makes sense given that both Batson and Eisenberg are psychologists interested in testing whether helping behavior produced by sympathy is altruistic. Maibom's definition of sympathy, by contrast, is closer to the one of Scheler and involves two emotions, one positive and one negative:

Person S sympathizes with person O when S feels sad for O as a result of believing or perceiving that something bad has happened to O, or S feels happy for O as a result of believing or perceiving that something good has happened to O (Maibom, 2017, p. 2)

However, she also seems open to include other feelings such as the ones Batson mentions (see Maibom, 2020), which suggests that her definition can potentially be widened to encompass those. Lastly, Darwall seems to define sympathy as one discrete emotion:

[Sympathy] is a feeling or emotion that (a) responds to some apparent threat or obstacle to an individual's good or wellbeing, (b) has that individual himself as object, and (c) involves concern for him, and thus for his wellbeing, for his sake. (Darwall, 1998, p. 261)

Like Batson and Eisenberg, Darwall restricts sympathy to situations where the other person's welfare is perceived as threatened, but he mentions only concern as a sympathetic feeling. Hence, while the authors discussed here agree that feeling sympathy consists in feeling an other-oriented emotion, they disagree on what it is and what emotions it involves.

Furthermore, it is not clear what it means for an emotion to be other-oriented. Batson explains that many emotions have self- and other-oriented versions (2011, p. 12). Several authors think that other-oriented versions of emotions have another person as objects (Darwall 261, Goldie 2000, 213–214). For instance, when we feel sorry for Alma who just lost her grandmother, our emotion is about her. This is, however, not sufficient to characterize other-oriented emotions. Admiration, contempt, schadenfreude, or mockery usually have another person as their objects but are not other-oriented in the way feeling sorry for another is. Other-oriented emotions are also felt on behalf of the other person and for her sake. How that translates into the structure of other-oriented emotions is unclear, but it implies that other-oriented emotions involve concern for the other person rather than a sharing of the other's experience (Coplan, 2004; Darwall, 1998, p. 274). Hence if we feel sorry for Alma who has just lost her grandmother because we care about her, our feeling is other-oriented; on the contrary, if we feel sorry because it means that Alma will not be coming to our party, it is not.

The idea that sympathy is a reaction of concern for the other explains why other-oriented emotions are “congruent with the perceived welfare of its object” (Batson, 2011, p. 11). Since we are concerned about the other person and want her to be well for her own sake (Darwall, 1998), we perceive her doing well as good and inversely. While we might be sad for Alma when she loses her grandmother, we will be happy for her when she finds the perfect apartment she was looking for.^5^ Similarly, since sympathy involves a desire for that person to be well, it motivates us to help that person when she is in need. When we feel sorry for Alma, we want her to be well for her own sake and therefore want to comfort her. It might motivate us to offer to spend time with her, to help her around the house, etc. Growing empirical evidence supports this claim (Batson, 2011; Eisenberg et al., 1989b; Leiberg et al., 2011). Batson and his team have conducted a number of studies showing that sympathy produces altruistic motivation (Batson, 2011).^6^ When the cost of helping is not too high, sympathetic subjects tend to act on their motivation to help even if there is an easy way to escape the situation. This suggests that sympathy is a pro-social attitude that plays an important role in our social lives.

However, we need to better understand the relation between sympathy and concern to specify how the former motivates helping behavior at the psychological level. It is not clear if concern is part of the emotional experience of feeling sorry or angry for another, occurring along with it, or causing it. Batson says that an emotion is other-oriented if the other person's “welfare is the focus of the emotion” (Batson, 2011, p. 12) but does not explain what this means. He argues, nevertheless, that valuing someone's welfare is an antecedent to feeling sympathy for that person (Batson, 2011, p. 33), implying that some form of concern is at least causally related to sympathy. In what follows, I undertake to offer a more precise account of sympathy and to elucidate what emotions it involves and how it relates to concern for another. I start by clarifying the structure of other-oriented emotions.

## The Structure of Other-Oriented Emotions

### What are Other-Oriented Emotions?

To understand better the structure of other-oriented emotions, let us compare an instance of other-oriented sadness with one of regular sadness. Imagine that Alma is sad because she has just been informed that her grandmother, whom she loved deeply, passed quietly in her sleep last night. Imagine also that Julia, a friend of Alma, has just learned about Alma's loss and feels sorry for her.^7^ At first sight, both emotions seem similar in terms of their particular objects and formal objects. The particular object of an emotion is what the emotion is about or directed at, while the formal object is the evaluative property that is implicitly attributed to the object in virtue of the emotion felt toward it (Scarantino & de Sousa, 2021). When I am afraid of going wingsuit flying, for example, the object of my emotion is going wingsuit flying and the formal object is dangerousness. It is because wingsuit flying appears dangerous to me that I react with fear to the thought of doing it. In the above example, both Alma and Julia are reacting to the death of Alma's grandmother, and both see it as a loss and therefore are sad. Despite those similarities, Alma's and Julia's experiences are quite different. Alma's sadness is most likely stronger than Julia's. But most importantly, Alma's emotion is about losing her own grandmother, while Julia's is about the loss of her friend. Both emotions are about the same event but have different particular objects.

It might therefore seem that regular emotions have particular objects that are related to oneself while other-oriented emotions have objects that are related to someone else. My fear that I go wingsuit flying is self-oriented whereas my fear of Zack going wingsuit flying is other-oriented. As we have seen above, this criterion does not work for emotions such as admiration, contempt, schadenfreude or mockery that can have another person as their object but are not felt for another person's sake. One might nevertheless think that there is something particular about these emotions and that the object criterion still applies to other emotions. However, if we look at those other emotions, we can see that this is not the case. We can be personally affected by an object that is related to someone else and conversely. For instance, Eloise might be worried for her children's sake that her plane crashes while on a business trip. In that case, what she is worried about is that she gets badly injured or even dies in a plane crash. The object of her emotion is directly related to her. However, she is worried *for* her children. It is out of concern for them that she is worried about getting injured in a plane crash because that possible event appears to her as a threat to her children's wellbeing. Conversely, Sirius might be afraid that his ex-partner gets hurt while wingsuit flying because he needs them to look after the children the following weekend. In that case, the object of his emotion is other-related—it is an event potentially happening to his ex-partner—but he does not feel worried for his partner's sake. He is worried about his ex-partner getting injured mainly because that would threaten his plans and not because he cares about them. These two examples suggest that the orientation of an emotion does not depend on its particular object, but rather on whom one is concerned about.

One might object that the description of the above examples is inaccurate and that the object of Eloise's worry is not her getting injured in a plane crash, but rather her children not being well taken care of. Similarly, the object of Sirius's fear would not be his ex-partner's having an accident, but his plans for the weekend falling out. In that case, these examples would fail to show that the object of an emotion is insufficient to determine its orientation. However, this objection fails. The fact that Eloise is worried that her children might not be well taken care of if something happens to her does not imply that she is not worried about getting injured in a plane crash for her children's sake as well. On the contrary, it is because she is concerned with her children's wellbeing that she also worries for them that she might get injured. At the time of boarding the plane, she might keep entertaining the possibility of the plane crashing and feel genuinely worried about it. Denying this would be like claiming that a law student is not worried about a coming test because what they are really afraid of is not getting their degree or not being able to become a lawyer. Although the student's desire to pass the test is instrumental to their further aims—which are objects of further worry—they are still very much concerned about passing the coming exam. Similarly, Eloise is worried about getting injured in a plane crash and Sirius about his ex-partner having an accident even if ultimately Eloise is mainly worried about her children's wellbeing and Sirius about being able to go away on a weekend.

One might then accept that Eloise is genuinely worried about getting injured because she is concerned about her children but still doubt that her worry is other-oriented. The fact that her concern for her children is the reason why she is worried about getting injured does not imply that her feeling is other-oriented. Perhaps it is her awareness of that connection and coexisting feeling of concern for her children's wellbeing that make Eloise's whole experience appear to her as being about her children. However, Eloise's concern for her children does not merely play a causal role in her worry about getting injured. Her situation is different from the one of being afraid of a friendly dog passing by because we were bitten as a child. In that case, the previous bad experience with a dog does not need to be part of the present emotional experience. In fact, we might not even remember having been bitten by a dog. By contrast, Eloise's concern for her children is, in some way, part of her emotional experience because her fear presents to her the possibility of getting injured in a plane crash as a threat to her children's wellbeing. Objects do not appear to us as dangerous in themselves. They appear to us as dangerous only to the extent that we perceive them as a threat to someone or something. For instance, peanuts are dangerous only for those who are allergic. If there are peanuts at a party but no one is allergic to them, we will not see them as dangerous for anyone and will not be afraid of anyone eating them. If we are allergic, however, we will be afraid of eating peanuts because they appear to us as dangerous for ourselves. For whom an object is dangerous is part of the emotional experience of fear; it gives it an orientation.^8^ For instance, let's say that I am babysitting my nephew who is running around in the living room on a small tricycle and that I am afraid that he runs into the console. I will experience this fear differently if he running into the console appears to me as a threat for himself, for the ceramic statue on the console or for me, because I would get in trouble with my sister. This will direct my attention, elicit different thoughts, activate different desires, and motivate me to react in different ways (catching my nephew vs. catching the statue). Hence, Eloise is afraid of the plane crashing because it appears to her as a threat to her children and Sirius is afraid of his ex-partner having an accident because it appears to him as a threat to his plans for the weekend.^9^ Similarly, Alma is sad because the death of her grandmother appears to her as a loss *for herself*, while for Julia, it appears as a loss for Alma. The object of the emotion is therefore evaluated with regard to another object that gives the emotion its orientation.

The orientation of an emotion is what some philosophers call the focus of concern (Ben-Ze’ev, 2001) or simply the focus of an emotion (Helm, 2007). While the object of an emotion is what is at the center of our attention during an emotional experience, the focus is what has “import to the subject that makes intelligible the evaluation implicit in the emotion” (Helm, 2007, p. 69). My fear of going wingsuit flying is intelligible in light of my concern for my own safety, while anger that my co-worker is late again for our weekly meeting is intelligible in light of my concern for my dignity and my desire to be respected. The formal object of the emotion informs us on how the object and the focus are related, and it is because of that relation that the object derives its import from the focus (Helm, 2007). Wingsuit flying appears to me as a threat to my own safety, while my co-worker's lack of punctuality appears to me as a disrespect to myself. If I did not care about my safety or my dignity, I would not react emotionally in those cases. Caring about someone or something^10^ disposes us to feel different emotions depending on how that person or thing fares in the world (Helm, 2007; Seidman, 2016). It disposes us to feel afraid for her if she is in danger, relieved when she is safe, sorry when she is sick, happy when she meets success, etc.

Other-oriented emotions can therefore be understood as emotions that have another person as their focus. Often, the focus of our emotions is ourselves, which is readily understandable from context. Therefore, we usually do not need to specify that we are feeling worried of not passing a test *for ourselves* and then that we are excited and relieved of having passed it *for ourselves*. When we feel other-oriented emotions, however, we often point out that we feel the emotion for another to stress that the emotion is not self-oriented.

### What Emotions can be Other-Oriented?

Interestingly, not all types of emotions can have another person as their focus. The core characteristics of particular emotions can constrain what kind of focus they typically have. The formal object of an emotion plays an important role in this. For instance, relief is felt when a threat to the focus of the emotion has disappeared or has been defeated. Hence, relief is typically only felt for something that we can value and that can be threatened. For example, we can feel relieved that the fire in the Amazon Forest has been extinguished, that the ball didn’t hit the precious ceramic statue, or that the child who fell into a well has been found alive, to the extent that they have some value to us and are the type of entities that can be threatened. However, a mathematical equation or a conceptual truth can hardly be the focus of relief. Similarly, admiration is an emotion felt for an object that displays a certain type of virtue or excellence (Roberts, 2003). This suggests that its focus is typically a type of virtue or excellence.^11^ This implies that another person is not an appropriate focus for admiration, and it is difficult to understand what it would mean to admire Mother Theresa for another person's sake.

For some emotions, it is rather straightforward whether they typically can have another person as focus or not. Sadness, anger, fear and joy and their close relatives (disappointment, indignation, worry, etc.)^12^ can easily be felt for others, unlike surprise, contempt and admiration which are not felt for anyone's sake. For other emotions such as guilt, shame, and pride, it is not so easy to determine if their focus can be a person. Guilt is often thought as the perception of a behavior of ours (action, thought, and desire) as having flouted a norm (Ben-Ze’ev, 2001; Deonna & Teroni, 2008). I feel guilty of not having recycled my bottle of water because I consider that doing so is a social or perhaps even a moral norm. This suggests that the typical focus of guilt is a (social, personal, cultural, moral, …) norm. However, it might also seem that when we feel guilty for having hurt someone we love, we do not feel it out of concern for the norm “do not hurt others” but out of concern for the person, we have hurt.

Furthermore, guilt, shame, and pride seem to have other characteristics that prevent them from being other-oriented. For example, shame can be understood as an object appearing as undermining one's respectability or worthiness (Roberts, 2003). Carlos is ashamed of the scar on his face because he perceives it as undermining his aesthetic value and therefore his overall value. We might think that the focus of shame could be someone else's value or image, but shame is usually considered a self-construal, i.e., it is only oriented toward ourselves (Ben-Ze’ev, 2001; Roberts, 2003; Deonna & Teroni, 2008). Thus, we can be ashamed of others only to the extent that it is “casting disgrace on oneself” either as an individual or as a group (a team, a nation, etc.) (Roberts, 2003, p. 227). The same might be the case with pride if it consists in seeing an object as confirming or enhancing our own worth (Roberts, 2003). In the case of guilt, even if its focus could be a person, its typical object further restricts the possibility of feeling it for others because guilt is about our own behavior. This might be why we typically do not feel guilty for others when they have breached a rule.

However, we seem to feel instances of pride or shame that are more appropriate to another's situation. It might be that those emotions have been mischaracterized and can have another person as focus. Another possibility is that they are in fact not genuinely other-oriented. They might be instances of empathy (see Section 4). For instance, the shame or embarrassment we feel when our friend is relating how he made a fool of himself might not be embarrassment *for* him, but embarrassment *with* him (see Section 4). It is also possible that we identify with the other person and that there is a self-other overlap. The pride that we feel for a child or a spouse might then be due to the fact that we see them as somehow part of ourselves. The fact that we typically do not feel pride, shame, and guilt for strangers although we can easily feel sad, concerned or happy for them supports these hypotheses. Whether pride and shame can be other-oriented is therefore not clear. Since the core characteristics of many emotions are still debated, further work is required to determine precisely what emotions can have another person as focus.

### Other-Oriented Emotions and Concern

We are now in a position to discuss how other-oriented emotions involve concern for others. To do that, it is important to clarify what “concern for others” means. Here, I do not understand it as an emotion of the type of worry, but rather as others being a matter of interest and a preoccupation. In other words, I understand it as caring about others. In this context, I will use the terms “valuing” “caring about” and “being concerned” interchangeably.

As we have seen, emotions arise when we perceive a significant change to something that is of import to us (Ben-Ze’ev, 2001; Goldie, 2000; Helm, 2007). Hence, our values dispose us to feel certain emotions depending on how they fare and thereby play a causal role in the elicitation of emotions.^13^ Julia's sadness for Alma informs us that she cares about Alma and that she is disposed to be worried for her if she is in danger, happy for her if she is well, etc. This is in line with Batson's claim that valuing is an antecedent of other-oriented emotions (2011). However, as explained above, our care for others does not merely cause emotions for others; it is part of the emotional experience itself. Liliana's preoccupation with her look is at the heart of her shame; Julia's preoccupation with Alma's wellbeing is part of her sadness for her. Julia appears to her as someone she values and who has suffered a loss. When we feel an emotion for someone, that person appears to us as a value to safeguard or promote. Helm says about self-oriented emotions that “in feeling an emotion, the import of one's situation impresses itself upon one” (Helm, 2007, p. 60). In other-oriented emotions, it is the other person's import that impresses itself upon us. It is in that sense that concern is part of the other-oriented emotional experience itself.

This enlightens why other-oriented emotions motivate helping actions. It is widely agreed that emotions motivate actions, although how they do so is still disputed (Frijda, 1986; Kenny & Kenny, 2003; Scarantino, 2014; Deonna & Teroni, 2012; Tappolet, 2016). Emotions motivate us to act in a way that is compatible with the evaluations they involve (Ben-Ze’ev, 2001, p. 61). For example, disgust can motivate us to move away from the disgusting object, anger to act in a way to bring justice, reparation or retaliation, and sadness to look for comfort. When we feel fear for someone else, we are concerned about them in the sense that this person's safety is presented to us as valuable and as needed to be safeguarded. As a result, we are motivated to prevent that event, for instance by grabbing our child's hand when crossing the road.^14^ Whether we act on that motivation will depend on other factors: we might not have the means to help that person, we might have a stronger motivation to help someone we value more, or the helping action might appear too costly for us. Nevertheless, we will be motivated to help and in situations where there is no competing motivation, we will act on that motivation.

### Other-Oriented Emotions for Strangers

How much we care about another person impacts how we react emotionally to what happens to them. Our emotional reactions are stronger for a loved one than for a distant acquaintance in the same situation. The more we care about someone, and the longer and more often we will also pay attention to that person (Helm, 2010) and therefore feel emotions for them. Similarly, we are willing to do more to help those we care about more. However, we do not need to care about another a lot to feel emotions for them. We usually value at least minimally the wellbeing of strangers if we do not have reasons to dislike them and we react emotionally to their perceived wellbeing (Batson, 2011). For instance, we will feel afraid for a motorcyclist who just had an accident and is lying on the road in front of our eyes and feel happy for a couple who just got engaged at the restaurant. Since we care about strangers very little by comparison with other people and other things in our lives, we might not react emotionally toward others if we are preoccupied with other things. Furthermore, when we do feel other-oriented emotions for them, our reaction will usually be less intense and short. We might never give another thought about the couple who got engaged after leaving the restaurant and as a result might never feel emotions for them again. Nevertheless, if we were to learn by chance that the motorcyclist is well after all, we would likely feel some relief for them, and we would feel sorry for the couple if we learned that their engagement was broken, even if very mildy. Hence, the dispositions involved with caring for others are weaker for people we care very little about, but they exist, nonetheless.

In this part, I have argued that other-oriented emotions are instances of emotions that have another person as focus. They involve concern for that person in the sense that we feel emotions for others that we value, and those emotions present us those people as a value to safeguard or promote. This is also why other-oriented emotions are pro-social and motivate altruistic actions when the other person is in need. Since sympathy is an other-oriented emotion, this explains why sympathy involves concern for the other and motivates helping actions. However, it is still not clear how sympathy relates to other-oriented emotions.

## Sympathy—How to Conceive of It?

We can now address the question of how to conceive of sympathy. We have seen that Batson, Darwall, Eisenberg, Coplan, and Maibom agree that sympathy is an other-oriented emotional phenomenon that is felt *for* another and that involves concern for that person. However, they disagree on what emotions constitute sympathy: Darwall seems to conceive of it as one discrete emotion (1998), others as happiness and sadness (Maibom, 2014; Scheler, 2017), as sorrow and concern (Coplan, 2004; Eisenberg & Eggum, 2009), and Batson as any other-oriented emotions felt for someone in need (2011). We might want to determine who is right.

However, doing so makes sense only if their disagreement is not a mere verbal dispute and that they really disagree about how to define the same phenomenon. Since the term “sympathy” has been used to refer to different emotional phenomena and what we have been calling “sympathy” is named differently depending on the disciplines, considerations pertaining to the common use of the term, its etymology, or how it has been used by philosophers in the past cannot help us discriminate between the possible conceptions of sympathy. This implies that to determine which conception of sympathy is the best or the correct one, we need to look at the thing itself. But what would that thing be? Darwall seems to conceive of sympathy as an emotion on its own, but it is not clear that such an emotion exists. If it does, it should be possible to describe its distinct phenomenology, formal object, and action readiness. Darwall does not provide such a detailed account and it is not obvious what it would look like. Batson's decision to restrict sympathy to other-oriented emotions for someone in need is merely practical and contextual and the other authors provide no justification for restricting sympathy to one or two emotions. Hence, it is not clear what the phenomenon of sympathy is and the decision to conceive of it as only one other-oriented emotion or a particular subset of them appears to be an arbitrary decision or at best a pragmatic one based on the needs of the research context.

This suggests that a good conception of sympathy is first and foremost a useful one. Therefore, I suggest conceiving of sympathy as referring to all instances of emotion that have another person as their focus. On this conception, sympathy is not a particular emotion. It involves feeling sorry for, happy for, worried for, angry for another and many other closely related feelings. This wide conception helps clarify the landscape of emotions. First, it enables us to distinguish those other-oriented emotions, which are pro-social, from the self-oriented ones, which are not. Other-oriented emotions motivate us to contribute to the wellbeing of others either by helping them when they are in need or by celebrating with them when something good happens to them, etc. Other-oriented emotions are also perceived both by the subject and others as expressions of concern and thereby help us create or sustain bonds with them. Other-oriented emotions thus play an important social role, and it is useful to have a concept to identify them. Second, this conception of sympathy provides us with a specific feature that helps us to distinguish other-oriented emotions from empathic ones. Although both types of emotional reactions are more appropriate to another's situation than our own (Hoffman, 2001) and can easily be confused, they have different structures and action tendencies (see next section). Hence, having a concept for each and understanding sympathy as referring to all other-oriented emotions is useful.

More restricted conceptions of sympathy as a subset of other-oriented emotions can still be useful in particular research contexts such as research on helping behavior and altruism. Otherwise, a more general conception of sympathy as referring to all instances of emotion that have another person as focus will be more useful to differentiate between different types of affective experience.

## Sympathy and Empathy—Distinguishing the Two

While there is extensive literature on empathy, the term has been used in many ways, giving rise to much debate and confusion. Today, the following phenomena are most often distinguished from one another:

- Affective empathy: feeling what another person is feeling while remaining aware of the self-other distinction and that one's feelings are the other's, so to speak (de Vignemont & Singer, 2006; Maibom, 2017)
- Emotional contagion: feeling what one or several people are feeling without being aware that we are feeling it because of them, e.g., becoming joyful when arriving at a party where people are having a good time (Darwall, 1998; Scheler, 2017; Stueber, 2010)
- Perspective-taking: imagining what another person is experiencing (other-imagining) or what we would experience if we were in another person's situation (self-imagining). (Coplan, 2011; Jackson et al., 2014; Lamm et al., 2007)
- Personal or empathic distress: becoming distressed when faced with another person's suffering (Decety, 2020; Singer & Klimecki, 2014)
- Sympathy: see previous sections

Some authors use the term “empathy” to refer to one of these processes or to several of them. For example, Hoffman as well as Preston and de Waal find that a wide conception of empathy that encompasses all of the above is more useful in their research contexts (Hoffman, 2001; Preston & Waal, 2002). In phenomenology, the concept of empathy is used in a complete different way and refers to a particular type of intentionality—a mode of consciousness like imagination or perception—that enables us to directly experience some mental states of others and to understand them (Gallagher, 2008; Schloßberger, 2020; Zahavi, 2001).

My interest here is in empathy conceived as affective empathy.^15^ It is especially interesting to compare it with sympathy because both are emotional experiences—unlike perspective taking—and are about another person and not ourselves—unlike emotional contagion and personal distress. Furthermore, both are more appropriate to another person's situation and can be instantiated through different emotions. Although this makes empathy and sympathy quite close, they are clearly distinct in two ways. First, empathy consists in feeling what another person is feeling and therefore requires some similarity between what the other person and we are experiencing.^16^ By contrast, we can feel a sympathetic emotion for someone whose experience is quite different, such as feeling worried for someone who is going wingsuit flying and who is excited about it. Second, empathy, unlike sympathy, does not involve concern for another, although empirical evidence shows that empathy often elicits sympathy (Batson, 2011; Lamm et al., 2007). The above account of the structure of sympathetic emotions is helpful to further untangle how an empathic and a sympathetic experience of the same emotion differ, also shedding light on why empathy does not directly motivate helping behavior.

As we have seen, when we feel sympathy, we react to what is happening to another out of concern for them. What emotion we feel depends on how we evaluate the other person's situation: we feel afraid for the other person if she appears to us as being in danger, angry if she appears to us as having been offended, etc. This implies that we are not looking at the other's situation from their own point of view, but from ours. Also, sympathetic emotions are *for* others in the sense that they always have another person as their focus.

By contrast, in empathy, we feel the other person's emotion as if we were that person or as if we were in her situation. In addition, we interpret that emotion as being what the other person is feeling, and project it onto them. We do not feel it from our own perspective, but from the perspective of the other person. This appears clearly when we feel empathy as a result of perspective-taking. If I take Alma's perspective and imagine that I am Alma and that I have lost my grandmother, I will feel sad about it. In that case, I feel sadness for “myself” for having lost “my” grandmother. But I remain aware that I am imagining being Alma and that the subject—the *I* of this imaginative experience—is Alma. This is also the case, although less clearly, when we feel empathy without perspective-taking, as a result of witnessing a person in a certain situation or believing that they are in that situation (Lamm et al., 2007; Maibom, 2018; Preston & Waal, 2002). When I meet with Alma and see how sad she is, I might automatically feel her sadness without having to imaginatively put myself in her shoes. Even in that case, however, my sadness is felt from Alma's perspective in the sense that it is sadness felt “for myself” for having lost “my” grandmother, where the *I* is still interpreted as Alma's. Hence, in empathy, we feel the emotion from the other person's perspective and not our own and we project that emotion on the other person. Empathy is other-oriented in the sense that the emotional experience is about another person, but in a different way than sympathy.

The emotion felt in empathy will often have the subject as focus, but not necessarily. When I empathize with Alma, I feel sadness for “myself,” but imagine that I am Alma. However, I might also empathize with a parent who is feeling worried for their child. In that case, I imagine that I am the parent feel the worry for the child. But regardless of whether the focus of the emotion is the self or another, it is not felt from our own perspective and the concern felt for “ourselves” or “the child” is not genuinely ours. This is what fundamentally distinguishes empathy from sympathy.

This difference in perspective explains why empathy and sympathy produce different motivations to act. When we empathize with another person's self-oriented emotion, that emotion presents ourselves as being in danger, offended, experiencing a loss, etc. and motivates us to safeguard or promote our own wellbeing. But since we are also aware that it is not our own emotion and that we are not in the other person's situation, that motivation remains idle.^17^ Hence, unlike sympathy, empathy does not directly motivate us to do anything, just like imagining that we are being chased by a lion does not motivate us to start running.

This does not imply that empathy has no impact on us. Putting ourselves in someone else's shoes can help us understand experientially what another person is feeling (Coplan, 2011). If that person is suffering or in danger and we value her, we might come to feel sympathy for her and/or be motivated to help her. Empathy then plays an epistemic role rather than a motivating one (Maibom, 2017). This is consistent with research showing that empathy often leads to sympathy (Batson, 2011; Eisenberg & Fabes, 1990; Singer & Klimecki, 2014). In fact, perspective-taking, which leads to empathy, is often used to elicit sympathy for strangers in need in lab experiments (Batson, 2011). Batson provides a causal explanation for the connection between empathy and sympathy:

Batson, Eklund et al. (2007, Experiment 2) found that increased valuing of another's welfare led to the spontaneous adoption of an imagine-other perspective, which in turn led to increased empathic concern [sympathy]. The downstream location of perspective taking explains why it can effectively induce empathic concern [sympathy] for someone in need. Even in the absence of prior valuing, it activates the valuing path. (Batson, 2011, p. 44)

It remains, however, unclear why perspective-taking and hence empathy retro-activated the valuing path, especially at the psychological level.

Interestingly, empirical evidence shows that empathy can also lead to personal distress (Batson et al., 1987; Decety & Lamm, 2011; Eisenberg et al., 1989a; Eisenberg & Fabes, 1990). Personal distress is a self-oriented reaction of distress to another's suffering (Singer & Klimecki, 2014) which motivates one to take care of oneself (Batson, 2011). Given what we have said about empathy, it is not surprising that it can lead to personal distress. Often, empathizing involves experiencing distress for ourselves that we project onto the other person. If the emotional experience is strong, we might start focusing on our feelings only and stop projecting it. We would thereby lose track of the fact that it is not our emotion that we are feeling (Decety & Lamm, 2011; Eisenberg & Eggum, 2009). Then, our self-oriented distress would motivate us to take care of ourselves since we would perceive ourselves as in need. This tends to happen when we are not good at self-regulating, i.e., at controlling how we focus and shift our attention (Eisenberg & Eggum, 2009). Evidence also shows that personal distress is more likely to arise if we imagine ourselves in the other person's situation rather than if we imagine what the other person is feeling (Lamm et al., 2007).

Empathy thus differs from sympathy in that the emotional experience is felt from the other person's point of view and projected onto them, while sympathy is felt from our own point of view and always has another person as focus. As a result, empathy does not directly motivate helping behavior but can indirectly do so by informing us on the other person's experience and leading to sympathy. However, empathy can also lead to personal distress motivating us to withdraw and take care of ourselves.

## Conclusion

In this paper, I have argued that sympathy should be understood as referring to all instances of emotion that have another person as focus. In other words, when we feel sympathy for another person, that person is presented to us as an object that we value and that is in danger, offended, experiencing a loss, etc., depending on our appraisal of that person's situation. As a result, when we perceive the other person as in need, we are readily motivated to help her. That understanding of sympathy clarifies its structure and explains why it can take the form of different emotions and involves concern for the other. I also contrasted sympathy with empathy and explained that empathy involves feeling an emotion from the other person's perspective and projecting that emotion onto them. Because the emotion experienced in empathy is not interpreted as “ours”, its motivational tendencies become idle. However, if that emotional experience is strong, we might lose the awareness that it is not “ours”, leaving us feeling distressed and needing to take care of ourselves. It remains nevertheless unclear how empathy leads to sympathy and more research is needed to understand how they are connected.

## Notes

1. What is called “sympathy” by philosophers is also called “empathic concern” (Batson, 2011), “compassion” (Singer & Klimecki, 2014) or even “empathy” (Hoffman, 2001) in other disciplines.

2. Scheler uses the term “sympathy” to refer to all these experiences. What I call “sympathy” here is Scheler's *Miteinanderfühlen*, which is a type of fellow-feeling (see Scheler, 2017; Schloßberger, 2016).

3. However, Scheler specified that sympathy does not imply that we get in a sorrowful or “joyful mood” ourselves (Scheler, 2017, p. 42). To see why that is, we need to understand Scheler's theory of feelings and the difference he made between different classes of feelings. This intricate matter is beyond the scope of the paper, but see Schloßberger, 2016 for a detailed account.

4. Although Batson uses the term “empathic concern” and not “sympathy”, it appears from his description that we are talking about the same emotional phenomenon: emotions felt *for* another, that are congruent with our perceived welfare of the other person and are felt out of concern for another. He also explicitly says that empathic concern is what Darwall calls “sympathy” (Batson, 2011). Empathic concern and sympathy have been often considered synonymous in recent literature (Darwall, 1998; Decety, 2020; Maibom, 2017; Singer & Klimecki, 2014).

5. However, although the valence of our other-oriented emotion will often match the one of the other person's experience, it is not necessarily the case since our evaluations of the situation might differ, as exemplified in the introduction. While Sarah might be very disappointed that she was not selected to join the first crew going to Mars, we might feel happy about it, knowing how dangerous the trip to Mars is.

6. Although the evidence collected by Batson and his colleagues is compelling, whether it conclusively shows that empathic concern produces altruistic motivation is still disputed. See Cialdini et al., 1987, 1997; Sober & Wilson, 1999.

7. In addition to her sympathetic sadness, Julia might also empathetically feel Alma's sadness, making it difficult to distinguish the two experiences. Furthermore, if because of her grandmother's death, Julia has to cancel a trip with Alma that she had been planning for a long time, Julia might feel sad about that as well, adding to the confusion. For the sake of the following analysis, let's first assume that there is nothing sad for Julia herself in this situation and let's focus on Julia's experience of sympathetic sadness alone. We can do that by mentally abstracting it from the empathetic sadness or by imagining what Julia is feeling when she considers Alma's situation without taking her perspective. If one finds that difficult, the example can be modified so that Alma is not feeling sad, but angry that her grandmother was taken away from her. In that case, Julia might still feel sympathetically sad for Alma, but will experience empathetic anger rather than sadness. The two experiences will then be more clearly distinguishable and comparable.

8. This understanding of emotions as having an orientation is similar to Helm's (2007). His notion of focus of an emotion is introduced in the next paragraph.

9. This view appears to be in line with Nanay's account of vicarious emotions (2013, 2018). It is, however, not clear if he would agree with the rest of my account of other-oriented emotions and of sympathy.

10. Since we can also value material and abstract objects for themselves such as works of art, sentimental objects, peace, democracy, it follows that they can be the focuses of our emotions as well. For instance, I can fear the visit of my nephews for the sake of my ceramic statue, and I can be relieved that antique statues have been spared by the war in Syria for their own sake. However, not all formal objects are appropriate for objects: it is difficult to imagine feeling embarrassed for the vase or angry for the statues. Although related, emotions for objects do not directly pertain to sympathy and will not be further explored here.

11. Admiration has also been described as a person-focused (as opposed to act-focused) emotion (Bell, 2011; Kauppinen, 2019). However, it does not seem that in this context the term “focus” is understood as an object of import that makes the emotional reaction intelligible.

12. The focus can be either a person herself or something related to her such as her safety, her image, her integrity, etc. to the extent that they ultimately show concern for that person.

13. For a discussion of whether and how dispositions can be causes, see Alvarez, 2017; Choi & Fara, 2018.

14. Whether this helping behavior constitutes altruism is a further question. It is possible that in such cases non-altruistic motivations such as guilt avoidance contribute more strongly to motivate helping behavior (Sober & Wilson, 1999). Our helping action might also not be altruistic if we care about others because we feel a sense of oneness with them (Cialdini et al., 1997).

15. Although I will refer to affective empathy as “empathy” for brevity purposes, I am not arguing that it is the best or the right definition of empathy. I am also not making any claims about the role that empathy as affective empathy plays in our social lives and whether it is our most fundamental or efficient way of learning about others’ mental states. For a discussion of these questions, see for example Gallagher, 2008; Goldman, 2002; Schloßberger, 2020; Zahavi, 2011.

16. There is no agreement on how close our experience needs to be to the other's in affective empathy and the limit is rarely clearly set. However, some perceived similarity is characteristic of affective empathy. At the very least, the empathizer has to think that her experience is more or less similar to what the other person is experiencing (Maibom, 2017).

17. This contrasts with the commonly held view that empathy (directly) motivates pro-social behavior (see for instance Hoffman 2001). However, when engaging with this literature, one should keep in mind that empathy is often not conceived as referring strictly to affective empathy. For a discussion regarding how affective empathy specifically might motivate altruistic and moral behavior in general, see Schramme, 2017.

## References

[bibr1-17540739221140404] Alvarez M. (2017). Desires, Dispositions and the Explanation of Action. In Lauria F. (Ed.), The Nature of Desire (pp. 119–136). Oxford University Press.

[bibr2-17540739221140404] Batson C. D. (2011). Altruism in humans. Oxford University Press.

[bibr3-17540739221140404] Batson C. D. Fultz J. Schoenrade P. A. (1987). Distress and empathy: Two qualitatively distinct vicarious emotions with different motivational consequences. Journal of Personality, 55(1), 19–39. 10.1111/j.1467-6494.1987.tb00426.x 3572705

[bibr4-17540739221140404] Bell M. (2011). Globalist attitudes and the fittingness objection. The Philosophical Quarterly, 61(244), 449–472. 10.1111/j.1467-9213.2010.684.x

[bibr5-17540739221140404] Ben-Ze’ev A. (2001). The subtlety of emotions. Bradford.

[bibr6-17540739221140404] Choi, S., & Fara, M. (2018). “Dispositions”, the stanford encyclopedia of philosophy (Spring 2021 Edition), Edward N. Zalta (ed.). https://plato.stanford.edu/archives/spr2021/entries/dispositions/

[bibr7-17540739221140404] Cialdini R. B. Brown S. L. Lewis B. P. Luce C. Neuberg S. L. (1997). Reinterpreting the empathy–altruism relationship: When one into one equals oneness. Journal of Personality and Social Psychology, 73(3), 481. 10.1037/0022-3514.73.3.481 9294898

[bibr8-17540739221140404] Cialdini R. B. Schaller M. Houlihan D. Arps K. Fultz J. Beaman A. L. (1987). Empathy-based helping: Is it selflessly or selfishly motivated? Journal of Personality and Social Psychology, 52(4), 749. 10.1037/0022-3514.52.4.749 3572736

[bibr9-17540739221140404] Coplan A. (2004). Empathic engagement with narrative fictions. The Journal of Aesthetics and Art Criticism, 62(2), 141–152. 10.1111/j.1540-594X.2004.00147.x

[bibr10-17540739221140404] Coplan A. (2011). Understanding empathy. In Coplan A. Goldie P. (Eds.), Empathy: Philosophical and psychological perspectives (pp. 3–18). Oxford University Press.

[bibr11-17540739221140404] Darwall S. (1998). Empathy, sympathy, care. Philosophical Studies, 89(2–3), 261–282. 10.1023/A:1004289113917

[bibr12-17540739221140404] de Vignemont F. Singer T. (2006). The empathic brain: How, when and why? Trends in Cognitive Sciences, 10(10), 435–441. 10.1016/j.tics.2006.08.008 16949331

[bibr13-17540739221140404] Decety J. (2020). Empathy in medicine: What it is, and how much we really need it. The American Journal of Medicine, 133(5), 561–566. 10.1016/j.amjmed.2019.12.012 31954114

[bibr14-17540739221140404] Decety J. Lamm C. (2011). Empathy versus personal distress: recent evidence from social neuroscience. In J. Decety, & W. Ickes (Eds), *The social neuroscience of empathy* (pp. 199–214). Cambridge: MIT Press.

[bibr15-17540739221140404] Deonna J. A. Teroni F. (2008). Differentiating shame from guilt. Consciousness and Cognition, 17(4), 1063–1400. 10.1016/j.concog.2008.02.002 18445530

[bibr16-17540739221140404] Deonna J. A. Teroni F. (2012). The emotions: A philosophical introduction. Routledge.

[bibr17-17540739221140404] Eisenberg N. Eggum N. D. (2009). Empathic responding: sympathy and personal distress. The Social Neuroscience of Empathy, 6(2009), 71–830. 10.7551/mitpress/9780262012973.003.0007

[bibr18-17540739221140404] Eisenberg N. Fabes R. A. (1990). Empathy: Conceptualization, measurement, and relation to prosocial behavior. Motivation and Emotion, 14(2), 131–149. 10.1007/BF00991640

[bibr19-17540739221140404] Eisenberg N. Fabes R. A. Miller P. A. Fultz J. Shell R. Mathy R. M. Reno R. R. (1989a). Relation of sympathy and personal distress to prosocial behavior: a multimethod study. Journal of Personality and Social Psychology, 57(1), 55. 10.1037/0022-3514.57.1.55 2754604

[bibr20-17540739221140404] Eisenberg N. Miller P. A. Schaller M. Fabes R. A. Fultz J. Shell R. Shea C. L. (1989b). The role of sympathy and altruistic personality traits in helping: a reexamination. Journal of Personality, 57(1), 41–67. 10.1111/j.1467-6494.1989.tb00760.x 2709301

[bibr21-17540739221140404] Frijda N. H. (1986). The emotions. Cambridge University Press.

[bibr22-17540739221140404] Gallagher S. (2008). Direct perception in the intersubjective context. Consciousness and Cognition, 17(2), 535–543. 10.1016/j.concog.2008.03.003 18442924

[bibr23-17540739221140404] Goldie P. (2000). The Emotions: A Philosophical Exploration. Oxford University Press.

[bibr24-17540739221140404] Goldman A. (2002). Simulation Theory and Mental Concepts. In Dokic J. Proust J. (Eds.), Simulation and Knowledge of Action (pp. 1–20). John Benjamins.

[bibr25-17540739221140404] Helm B. W. (2007). Emotional Reason: Deliberation, Motivation, and the Nature of Value. Cambridge University Press.

[bibr26-17540739221140404] Helm B. W. (2010). Love, Friendship, and the Self: Intimacy, Identification, and the Social Nature of Persons. Oxford University Press. http://www.oxfordscholarship.com/view/10.1093/acprof:oso/9780199567898.001.0001/acprof-9780199567898

[bibr27-17540739221140404] Hoffman M. L. (2001). Empathy and Moral Development: Implications for Caring and Justice. Cambridge University Press.

[bibr28-17540739221140404] Hume D. (1738). A Treatise of Human Nature. Oxford University Press.

[bibr29-17540739221140404] Jackson S. M. Hillard A. L. Schneider T. R. (2014). Using implicit bias training to improve attitudes toward women in STEM. Social Psychology of Education, 17(3), 419–438. 10.1007/s11218-014-9259-5

[bibr30-17540739221140404] Kauppinen A. (2019). Ideals and Idols: On the Nature and Appropriateness of Agential Admiration. In Archer A. Grahlé A. (Eds.), The Moral Psychology of Admiration (pp. 29–44). Rowman and Littlefield.

[bibr31-17540739221140404] Kenny D. A. Kenny A. (2003). Action, Emotion and Will (2nd ed.). Routledge. 10.4324/9780203711460

[bibr32-17540739221140404] Lamm C. Batson C. D. Decety J. (2007). The neural substrate of human empathy: Effects of perspective-taking and cognitive appraisal. Journal of Cognitive Neuroscience, 19(1), 42–58. 10.1162/jocn.2007.19.1.42 17214562

[bibr33-17540739221140404] Leiberg S. Klimecki O. Singer T. (2011). Short-Term compassion training increases prosocial behavior in a newly developed prosocial game. PLOS ONE, 6(3), e17798. 10.1371/journal.pone.0017798 21408020 PMC3052380

[bibr34-17540739221140404] Maibom H. (2014). Introduction: (Almost) everything you ever wanted to know about empathy. In H. Maibom (Ed.), *Empathy and morality* (pp. 1–40). New York: Oxford University Press.

[bibr35-17540739221140404] Maibom H. (2017). Affective Empathy. In Maibom H. (Ed.), The Routledge Handbook of Philosophy of Empathy (pp. 22–32). Routledge.

[bibr36-17540739221140404] Maibom H. (2018). Self-Simulation and empathy. In N. Roughley, & T. Schramme (Eds), Forms of fellow feeling: Empathy, sympathy, concern and moral agency (pp. 109–132). Cambridge: Cambridge University Press.

[bibr37-17540739221140404] Maibom H. (2020). Empathy. Routledge.

[bibr38-17540739221140404] Nanay B. (2013). Between perception and action. Oxford University Press.

[bibr39-17540739221140404] Nanay B. (2018). Catharsis and vicarious fear. European Journal of Philosophy, 26(4), 1371–1380. 10.1111/ejop.12325

[bibr40-17540739221140404] Preston S. D. Waal F. B. M. de. (2002). Empathy: Its ultimate and proximate bases. Behavioral and Brain Sciences, 25(1), 1–20. 10.1017/S0140525X02000018 12625087

[bibr41-17540739221140404] Roberts R. C. (2003). Emotions: An essay in aid of moral psychology. Cambridge University Press.

[bibr42-17540739221140404] Scarantino A. (2014). The motivational theory of emotions. In Moral psychology and human agency: Philosophical essays on the science of ethics (pp. 156–185). Oxford University Press. 10.1093/acprof:oso/9780198717812.003.0008

[bibr43-17540739221140404] Scarantino, A., & de Sousa, R. (2021). Emotion, the stanford encyclopedia of philosophy (Summer 2021 Edition), Edward N. Zalta (ed.). https://plato.stanford.edu/archives/sum2021/entries/emotion/

[bibr44-17540739221140404] Scheler M. (2017). The Nature of Sympathy. Routledge. 10.4324/9781315133348

[bibr45-17540739221140404] Schloßberger M. (2016). The varieties of togetherness: Scheler on collective affective intentionality. In Salice A. Schmid H. B. (Eds.), The phenomenological approach to social reality (pp. 173–195). Springer.

[bibr46-17540739221140404] Schloßberger M. (2020). Beyond empathy: Compassion and the reality of others. Topoi, 39(4), 771–778. 10.1007/s11245-019-09636-7

[bibr47-17540739221140404] Schramme T. (2017). Empathy and altruism. In The routledge handbook of philosophy of empathy (pp. 203–214). Routledge.

[bibr48-17540739221140404] Seidman J. (2016). The unity of caring and the rationality of emotion. Philosophical Studies, 173(10), 2785–2801. 10.1007/s11098-016-0637-z

[bibr49-17540739221140404] Singer T. Klimecki O. M. (2014). Empathy and compassion. Current Biology, 24(18), R875–R878. 10.1016/j.cub.2014.06.054 25247366

[bibr50-17540739221140404] Slote M. A. (2007). The ethics of care and empathy. Routledge.

[bibr51-17540739221140404] Smith A. (2010). The Theory of Moral Sentiments. Penguin.

[bibr52-17540739221140404] Sober E. Wilson D. S. (1999). Unto Others: The Evolution and Psychology of Unselfish Behavior. Harvard University Press.

[bibr53-17540739221140404] Stueber K. (2010). Rediscovering Empathy: Agency, Folk Psychology, and the Human Sciences. MIT Press.

[bibr54-17540739221140404] Tappolet C. (2016). Emotions, Values, and Agency. Oxford University Press. 10.1093/acprof:oso/9780199696512.001.0001

[bibr55-17540739221140404] Zahavi D. (2001). Beyond empathy. Phenomenological approaches to intersubjectivity. Journal of Consciousness Studies, 8(5–6), 151–167.

[bibr56-17540739221140404] Zahavi D. (2011). Empathy and direct social perception: A phenomenological proposal. Review of Philosophy and Psychology, 2(3), 541–558. 10.1007/s13164-011-0070-3

